# Long‐term trends of epibionts reflect Mediterranean striped dolphin abundance shifts caused by morbillivirus epidemics

**DOI:** 10.1111/1365-2656.70216

**Published:** 2026-02-06

**Authors:** Sofía Ten, Gates Dupont, Juan Antonio Raga, Andy P. Dobson, Francisco Javier Aznar

**Affiliations:** ^1^ Marine Zoology Unit, Cavanilles Institute of Biodiversity and Evolutionary Biology Universitat de València Valencia Spain; ^2^ Department of Ecology & Evolutionary Biology Princeton University Princeton New Jersey USA

**Keywords:** cetacean, dolphin morbillivirus, epibiont, GAM, host–parasite interactions, marine mammal epidemiology, population dynamics, SIR model

## Abstract

1. Since 1990, Mediterranean striped dolphins, *Stenella coeruleoalba*, have suffered two mass mortality events caused by the dolphin morbillivirus (DMV), but the population‐level impact is unknown because abundance estimates are spatio‐temporally sparse.
2. This study investigates whether data from epibionts of striped dolphins—the barnacle *Xenobalanus globicipitis*, the cyamid *Syncyamus aequus*, and the copepod *Pennella balaenoptera*, with different life cycles and degrees of specificity—could provide indirect evidence on host population dynamics. To address this question, we combined empirical and theoretical approaches.
3. First, we used Generalized Additive Models (GAMs) to examine occurrence trends of the three epibiotic species over the period 1980–2023 for both striped dolphins and other sympatric cetacean species that did not suffer DMV outbreaks.
4. Second, we developed a two‐step theoretical modeling approach to investigate the epidemiology of these DMV outbreaks (SIR model) and to link dolphin population abundance shifts with the epibiont trends observed empirically (mechanistic model). The SIR model provided coarse estimates of the impact of DMV on the striped dolphin population under two scenarios with varying virus‐induced mortality and duration of the infectious period. These estimates were then used to simulate the effect of dolphin population shifts on its epibionts through mechanistic models.
5. Models indicated that DMV‐induced shifts in striped dolphin population dynamics have cascading effects on the population abundance of *X. globicipitis* and *S. aequus*, whereas the population of the less host‐specific *P. balaenoptera* was unaffected. Together, long‐term trends in the occurrence of host‐specific epibionts can serve as an indicator of host abundance shifts.

## Graphical abstract

This original research piece demonstrates, through empirical and theoretical modelling approaches, that the epibionts of striped dolphins indicate dolphin abundance shifts caused by epidemics of dolphin morbillivirus (DMV). In addition, we provide the first SIR model to investigate the epidemiology of DMV in western Mediterranean striped dolphins. Further epidemiological data will improve the predictive power of these models.

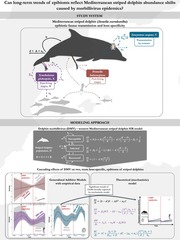

## Resumen

1. Desde 1990, los delfines listados del Mediterráneo, *Stenella coeruleoalba*, han sufrido dos episodios de mortalidad masiva causados por el morbillivirus de los delfines (DMV); sin embargo, el impacto a nivel poblacional es desconocido debido a que las estimaciones de abundancia son espacial y temporalmente escasas.
2. Este estudio investiga si los datos procedentes de los epibiontes de los delfines listados—el cirrípedo *Xenobalanus globicipitis*, el ciámido *Syncyamus aequus* y el copépodo *Pennella balaenoptera*, con diferentes ciclos de vida y grados de especificidad—podrían aportar evidencia indirecta sobre la dinámica poblacional del hospedador. Para abordar esta cuestión, combinamos enfoques empíricos y teóricos.
3. En primer lugar, utilizamos Modelos Aditivos Generalizados (GAMs) para examinar las tendencias de ocurrencia de las tres especies epibiontes durante el período 1980–2023, tanto en delfines listados como en otras especies de cetáceos simpátricos que no sufrieron brotes de DMV.
4. En segundo lugar, desarrollamos un enfoque teórico de modelización en dos etapas para investigar la epidemiología de estos brotes de DMV (modelo SIR) y vincular los cambios en la abundancia poblacional de los delfines con las tendencias de los epibiontes observadas empíricamente (modelo mecanicista). El modelo SIR proporcionó estimaciones generales del impacto del DMV en la población de delfines listados bajo dos escenarios con distinta mortalidad inducida por el virus y diferente duración del período infeccioso. Estas estimaciones se utilizaron posteriormente para simular el efecto de los cambios poblacionales de los delfines sobre sus epibiontes mediante modelos mecanicistas.
5. Los modelos indicaron que los cambios en la dinámica poblacional de los delfines listados inducidos por el DMV tienen efectos en cascada sobre la abundancia poblacional de *X. globicipitis* y *S. aequus*, mientras que la población del epibionte menos específico del hospedador, *P. balaenoptera*, no se ve afectada. En conjunto, las tendencias a largo plazo en la ocurrencia de epibiontes específicos del hospedador pueden servir como un indicador de cambios en la abundancia del hospedador.

## 1 INTRODUCTION

### 1.1 Western Mediterranean striped dolphin morbillivirus

#### 1.1.1 Outbreaks

The striped dolphin, *Stenella coeruleoalba* (Meyen, 1833), is the most abundant cetacean in the Mediterranean Sea (Gnone et al., 2023). As for other cetaceans, this species suffers from several anthropogenic threats, including chemical pollution, bycatch and prey depletion by overfishing (Aguilar, 2000; Bearzi et al., 2006; Izquierdo‐Serrano et al., 2022; Lauriano et al., 2014). In addition, dolphin morbillivirus (DMV)—a strain of cetacean morbillivirus, CeMV (Van Bressem et al., 2014)—has caused two mass mortalities in western Mediterranean striped dolphins (WMSDs) since it was first reported in 1990 (Domingo et al., 1990). DMV can cause fatal pneumonia or encephalitis, as well as severe immunosuppression that may lead to death due to opportunistic infections (see Van Bressem et al., 2014 and references therein).

The first outbreak was detected along the Spanish coast in July 1990 and spread eastward until 1992. This epizootic affected the adults mostly, with additional high rates of calf mortality due to maternal loss (Aguilar & Raga, 1993). The precise death toll of this epidemic could not be ascertained (Aguilar & Raga, 1993; Forcada et al., 1994; Van Bressem et al., 2014), and indirect measures of the impact of DMV were, therefore, used. On the one hand, mean pod size was ca. 25 and 7 before and during the 1990 outbreak, respectively, and 15–24 individuals in later years (Aguilar, Borrell, et al., 1991; Aguilar, Pastor, & Forcada, 1991; Forcada et al., 1994 and references therein; de Gómez Segura et al., 2006; Forcada & Hammond, 1998; Gannier, 2005; Paradell et al., 2023). On the other, surveys conducted in the western Mediterranean about 15 years later indicated that the abundance was 43% greater than after the epizootic, suggesting that the population had, at least in part, recovered (Cotté et al., 2010). Also, DMV was not immunohistochemically detected in dolphins stranded throughout the interepizootic period (Soto, Alba, et al., 2011; Soto, Alegre, et al., 2011).

In the summer of 2007, a different DMV strain caused a second epidemic in WMSDs and also caused fatalities in long‐finned pilot whales, *Globicephala melas* (Traill, 1809) (Bellière et al., 2011; Raga et al., 2008). After the population recovery from the previous epizootic, a high density of striped dolphins and a large proportion of susceptible individuals likely favoured the development of this new epidemic (Raga et al., 2008). The mortality was likely lower than in the previous outbreak; although, again, the total death toll could not be estimated (Van Bressem et al., 2014). During this second outbreak, most casualties were from juveniles, presumably because a high proportion of adults had retained immunity from the preceding epizootic (Raga et al., 2008).

In March–April 2011, there was an abnormally high number of strandings of striped dolphins on the Valencian coast (Spain); these were mostly juveniles and were DMV‐positive (Rubio‐Guerri et al., 2013). There is no evidence of DMV‐caused epidemics in other cetacean species resident in the Mediterranean Sea; DMV has caused occasional deaths in these species, except for an increased mortality of Mediterranean long‐finned pilot whales in 2006–2007 (e.g. Beffagna et al., 2017; Mazzariol et al., 2017; Van Bressem et al., 2014; Wierucka et al., 2014).

#### 1.1.2 Indirect measures of outbreak population impact

Understanding the impact of DMV epidemics on the WMSD population is challenging. First, the epidemiology of infection is complex; several factors may increase the severity of DMV and favour transmission, including high population density (de Gómez Segura et al., 2006; Raga et al., 2008), loss of herd immunity (Van Bressem et al., 2014) or genetic, environmental and anthropogenic factors—for example inbreeding (Valsecchi et al., 2004), water temperature (Aguilar & Raga, 1993) or high contaminant loads (Aguilar & Borrell, 1994; Borrell & Aguilar, 1991). Second, data on population size before and after the epizootics are scanty and not easily comparable, as surveys on WMSDs have used different spatial coverage and methodologies (i.e. small boats, large research vessels or aircraft; Arcangeli et al., 2016; Boisseau et al., 2010; Cotté et al., 2010; de Gómez Segura et al., 2006; Forcada & Hammond, 1998; Paradell et al., 2023). Stranded animals could be a valuable additional source of population data, especially to estimate the magnitude of the mortality events. However, bias could also be significant because the sample of animals washed ashore is typically small with respect to the wild population and is mainly composed of individuals from specific age classes or health conditions (Epperly et al., 1996; Moore et al., 2020).

During the 1990 epizootic, stranded WMSDs showed a significantly higher prevalence of two common epibiotic crustaceans: the copepod *Pennella balaenoptera* (Koren & Danielssen, 1877) and the barnacle *Xenobalanus globicipitis* (Steenstrup, 1852). It is likely that these hosts were immunosuppressed and suffered from impaired mobility due to DMV, thus increasing their susceptibility to colonization by epibionts (Aznar et al., 1994, 2005). This raises the question of whether long‐term trends of epibiont infections could also provide insight into the impact of DMV outbreaks on WMSDs. Anderson and May (1978) developed a foundational theoretical framework to describe the relation between host–parasite population dynamics, including the effect of host density on parasite transmission or the time‐lagged fluctuations in host–parasite populations (see also, e.g. Arneberg et al., 1998; Hansen & Poulin, 2006; May & Anderson, 1978).

### 1.2 Study aim

This study investigates the cascading effects of a microparasite (i.e. DMV) on its host macroparasites (i.e. the epibionts of striped dolphins) and the indicator potential of the latter for studying host (dolphin) population trends. To address this overarching question, we combine an empirical analysis of long‐term data with a theoretical modelling framework, allowing us to link observed epibiont trends with the demographic impact of DMV outbreaks.

First, we investigate long‐term temporal trends in the occurrence of each of the epibiotic species on WMSDs with empirical data. We hypothesized that severe mortalities in the WMSD population could cause declines in the population of epibionts, especially of *X. globicipitis* and *S. aequus*, because (i) these species depend exclusively on cetaceans (and *S. aequus* mostly on striped dolphins) and (ii) WMSDs may represent over 80% of the cetaceans living in the western Mediterranean (see Gnone et al., 2023; Raga & Pantoja, 2004). To separate the effect of DMV on striped dolphins from broader ecological changes, we also investigate the temporal trends of epibionts from other sympatric cetacean species that were not affected by DMV epidemics.

Second, we develop a theoretical model to shed light on the epidemiology of DMV outbreaks affecting WMSDs and to link dolphin population abundance shifts with changes in epibiont populations (as observed empirically). We first build a Susceptible‐Infected‐Recovered (SIR) epidemic model to estimate the impact of DMV on the WMSD population under two possible scenarios. Then, we simulate the dolphin population loss estimated from each scenario in an epibiont‐dolphin mechanistic model to assess the cascading effects on the epibiont populations.

### 1.3 Study system

To investigate whether, and how, epibionts can assist in elucidating the effects of DMV mortality on the WMSD population, we focus on the infection parameters of three obligate epibiotic species that are typically found on WMSDs and that have different life cycles and degrees of host specificity: the mesoparasitic copepod *P*. *balaenoptera*, the commensal barnacle *X*. *globicipitis* and the ectoparasitic whale louse *Syncyamus aequus* (Lincoln & Hurley, 1981).

The mesoparasitic copepod *Pennella balaenoptera*, now considered a member of the species complex *P. filosa* (Fraija‐Fernandez et al., 2018; Suyama et al., 2021; Ten et al., 2025), reproduces on a fish or cephalopod intermediate host (e.g. Arroyo et al., 2002) and inseminated females then seek a cetacean or large oceanic fish on which they attach, subsequently producing and releasing the eggs. Females of *P. balaenoptera* have been reported in all Mediterranean cetaceans, but also occur on a broad array of fish hosts (Ten et al., 2022). In contrast, the commensal barnacle *Xenobalanus globicipitis* is specific to cetaceans; it attaches to the fins, where it reproduces and releases larvae that will attach to another cetacean within days or a few weeks (Dreyer et al., 2020). This barnacle has been reported in all the cetacean species found in the Mediterranean (Ten et al., 2022). Finally, the cyamid *Syncyamus aequus* is an ectoparasitic amphipod almost exclusively found on striped dolphins, with sparse records on common dolphins, *Delphinus delphis* L. (Ten et al., 2022). This parasite lacks a free‐living stage and is transmitted by bodily contact between hosts (Fransen & Smeenk, 1991; Pfeiffer, 2009).

## 2 MATERIALS AND METHODS

### 2.1 Data collection

**FIGURE 1:**
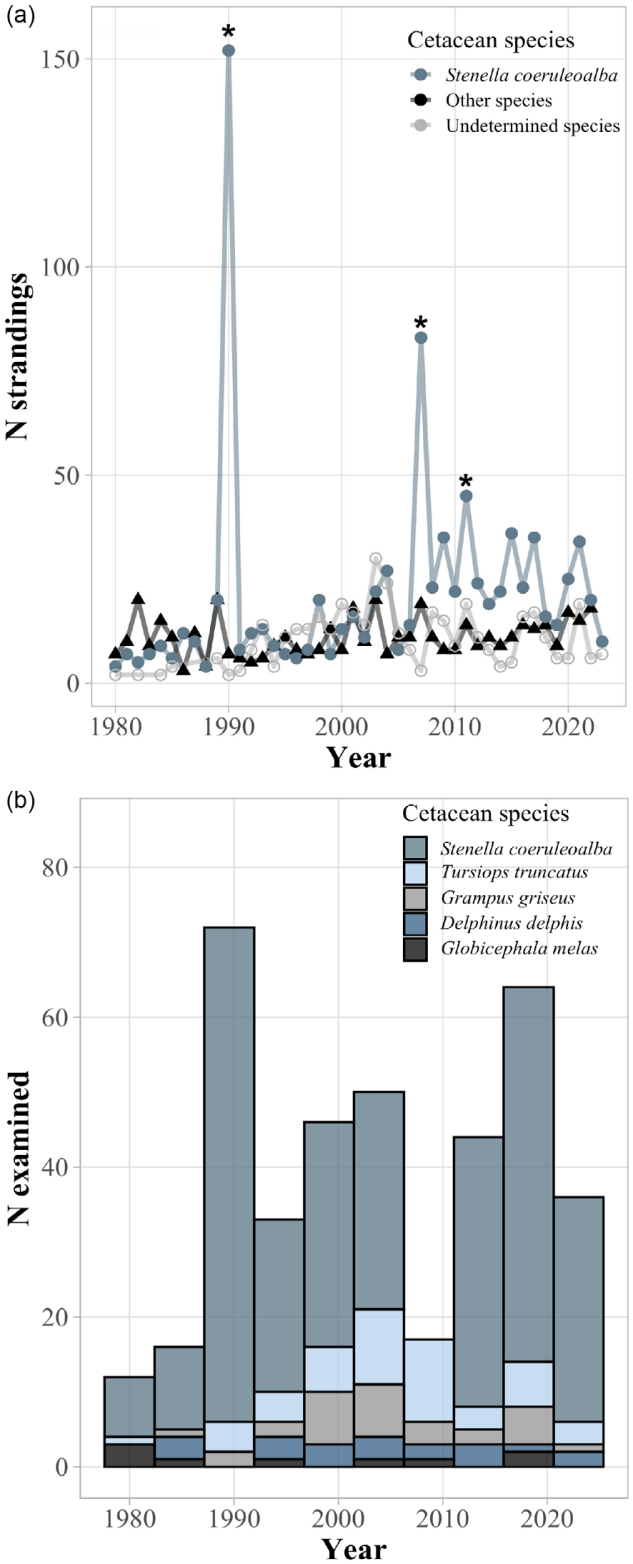
Cetacean strandings along the Valencian coast of Spain, western Mediterranean, in the period 1980–2023. (a) Total number, N, of strandings of striped dolphins (*Stenella coeruleoalba*) and other cetacean species, with asterisks indicating the three dolphin morbillivirus mortalities. (b) N of individuals of each cetacean species examined in this study.

A total of 1815 cetaceans were found stranded along the central Mediterranean coast of Spain, from 40°31.50′ N, 0°31.00′ E to 37°50.70′ N, 1°37.50′ W, between 1980 and 2023 (Figure 1a; MEDACES, 2024). Dead cetaceans with low signs of decomposition (code 2–3 sensu, e.g. Geraci & Lounsbury, 2005) were necropsied and data and samples of their epibionts were gathered by members of the Marine Zoology Unit of the University of Valencia. Epibionts were photographed, identified, counted and a subsample stored (in ethanol or frozen) during the necropsy. Permission and funding to collect stranded dolphins were given by the Wildlife Service of the Valencian Regional Government, Spain. No further permits related to ethical approval were required.

In this study, we analysed the epibiotic data from a total of 462 cetaceans. In addition to 355 striped dolphins (the target species), we included another 107 cetaceans: bottlenose dolphins, *Tursiops truncatus* (Montagu, 1821) (*N* = 48); Risso's dolphins, *Grampus griseus* (G. Cuvier, 1812) (*N* = 30); common dolphins, *Delphinus delphis* (*N* = 20); and long‐finned pilot whales, *Globicephala melas* (*N* = 9) (Figure 1b). We selected cetacean species that are well represented throughout the study period to avoid sampling bias when exploring temporal trends (i.e. periods enriched with a particular cetacean species) since epibiotic infection patterns may differ between cetacean species (due to, e.g. their bathymetric distribution; Fraija‐Fernandez et al., 2018; Table 1).

**TABLE 1:** Infection parameters of three epibiotic species on cetaceans stranded along the central Mediterranean coast of Spain.

Cetacean species (*N*)	Period	*Xenobalanus globicipitis*		*Pennella balaenoptera*		*Syncyamus aequus*	
		P (*N*) [95% CI]	MI (*N*) [95% CI]	P (*N*) [95% CI]	MI (*N*) [95% CI]	P (*N*) [95% CI]	MI (*N*) [95% CI]
Striped dolphin (355)	1990 epidemic	56.4 (55) [42.7–69.2]	9.9 (20) [6.1–15.8]	40.4 (57) [27.9–53.5]	5.3 (21) [3.3–8.3]	22.2 (54) [12.7–35.1]	3.8 (6) [2.3–6.0]
	2007 epidemic	47.1 (17) [25.3–71.3]	17 (1) —	11.8 (17) [2.1–35.0]	5 (1) —	31.2 (16) [13.2–56.4]	NA
	2011 mortality	37.5 (8) [11.1–71.1]	NA	0 (9) —	0	25.0 (8) [4.6–63.5]	NA
	Other periods (1980–2023)	38.8 (263) [33.1–44.9]	9.2 (53) [6.2–15.5]	17.5 (269) [13.3–22.5]	34.2 (39) [15.5–71.0]	26.9 (260) [21.7–32.7]	6.1 (42) [4.2–9.9]
Bottlenose dolphin (48)	1981–2022	27.9 (43) [15.9–43.0]	8.3 (37) [2.8–23.3]	8.7 (46) [3.0–20.4]	7 (1) —	—	—
Risso's dolphin (30)	1987–2022	53.3 (30) [34.8–70.2]	20.8 (4) [8.3–30.0]	36.7 (30) [21.3–55.1]	29.2 (9) [10.1–61.2]	—	—
Common dolphin (20)	1983–2022	60.0 (20) [37.2–79.1]	13.2 (4) [6.0–25.2]	30.0 (20) [14.0–52.5]	7.2 (5) [2.4–13.8]	15.0 (20) [4.2–37.2]	6.5 (2) [2.0–6.5]
Long‐finned pilot whale (9)	1982–2018	60.0 (5) [18.9–92.4]	4.7 (5) [2.0–7.3]	28.6 (7) [5.3–65.9]	102.0 (7) [5.0–102.0]	—	—

*Note*: For striped dolphins, parameters are grouped by time periods, that is dolphin morbillivirus mortalities versus other periods. NAs are shown when no intensity values were available.

Abbreviations: CI, confidence interval; MI, mean intensity; N, number of dolphins available; P, prevalence (%).

### 2.2 Infection parameters

We followed Reiczigel et al. (2019) to calculate, for each epibiotic species, the prevalence (i.e. proportion of infected hosts) and mean intensity (mean no. of individuals per infected dolphin); 95% confidence intervals (CI) were estimated, respectively, with Sterne's method and bias‐corrected and accelerated bootstrap (BCa) with 10,000 replications.

For the striped dolphins, we calculated these parameters separately for dolphins stranded during the DMV mortalities (i.e. June–December 1990, July–October 2007 and March–April 2011) and periods not affected by these mortalities. The prevalence and mean intensity during each outbreak were compared with the non‐epidemic periods with Unconditional Exact Tests and bootstrap two‐sample *t*‐tests with 10,000 replications, respectively (Reiczigel et al., 2019).

### 2.3 Long‐term trends in epibiont occurrence: Empirical data

Binomial Generalized Additive Models (GAMs) were used to model the likelihood of occurrence of each epibiotic species throughout the study period. In the case of *P. balaenoptera* and *X. globicipitis*, models were run separately for striped dolphins (*N* = 355) and for all the other odontocetes combined (to ensure a sufficient sample size, *N* = 107). The occurrence of *S. aequus* was modelled only for striped dolphins because it is seldom found in other cetaceans (Table 1). We first used the whole dataset to evaluate the effect of the DMV outbreaks on epibiosis. Then, we refitted the GAMs excluding DMV data (i.e. June–December 1990, July–October 2007 and March–April 2011) for assessing the robustness of the temporal trend.

GAMs included the presence/absence of each epibiotic species as the response variable, ‘year’ and ‘host length’ as smooth terms and ‘host sex’ (i.e. female, male or unknown) as a fixed effect. Host length and sex were included in the models to avoid potential confounding effects, i.e. differences in susceptibility to epibiosis between age classes or sexes. To investigate possible seasonal patterns, we also added the smooth term ‘season’ (with a cyclic cubic spline, ‘cc’) and the interaction ‘season: year’ (since the 1990 and 2007 epidemics peaked during the summer). We examined pairwise concurvity values to check for nonlinear collinearity (concurvity) between the smoothed terms of each GAM, with 0 values indicating no issues and 1 indicating a total lack of identifiability (Wood, 2017). All values were generally <0.1, except for ‘year’ and the interaction ‘season:year’ (i.e. ca. 0.8). Gamma was set to 1.4 to control overfitting (Wood, 2006). Backward model selection was based on Akaike's Information Criterion (Akaike, 1973) adjusted for small sample sizes (AICc; Hurvich & Tsai, 1989), considering models with ∆AICc >4 to be substantially less supported (see, e.g. Burnham & Anderson, 2002). From the better supported models, with ∆AICc <2, those with higher deviance explained were selected for graphical representation.

### 2.4 DMV epidemiology and cascading effects on epibionts: Theoretical modelling

We developed a two‐step modelling approach to capture the system dynamics, including the impact of DMV on western Mediterranean striped dolphins, WMSDs (SIR model) and then the subsequent effects on the population of the epibionts (epibiont‐dolphin mechanistic model), assessing the reliability of long‐term data on epibionts as indicators of hosts' abundance shifts.

#### 2.4.1 Susceptible‐infected‐recovered (SIR) model

**FIGURE 2:**
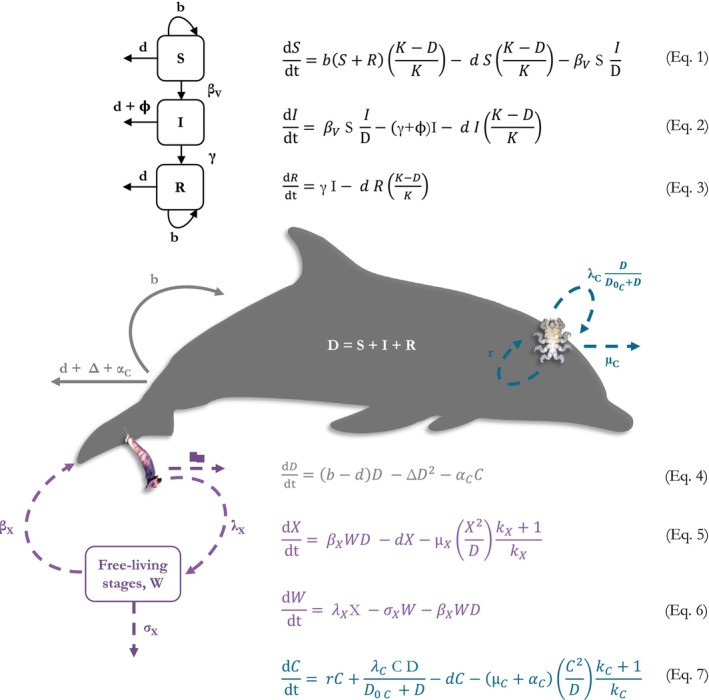
Two‐step model framework for investigating the impact of morbillivirus outbreaks on striped dolphin (*Stenella coeruleoalba*) abundance (*D*) and cascading effects on the populations of the epibionts *Xenobalanus globicipitis* (*X*, purple) and *Syncyamus aequus* (*C*, blue). Arrowed lines (solid, dolphins; dashed, epibionts) indicate biomass fluxes, with per capita rates shown on the side. Diagrams not to scale.

We first used an SIR model (Kermack & McKendrick, 1927) adapted to the study system (Equations 1–4 in Figure 2) to investigate the effect of DMV outbreaks on striped dolphin abundance. Model parameters (Table S1) were obtained from the literature, estimated from the dataset or adjusted to the expected trend based on the available data, that is at least two severe outbreaks, in 1990 and 2007 (see the Introduction), with a population abundance estimate of 15,778 (95% CI = 10,940–22,756) in 2001–2003 in the study area (de Gómez Segura et al., 2006).

The striped dolphin population (*D*) was considered to be composed of susceptible (*S*), infected (*I*) and recovered (*R*) individuals; hence *D* = *S* + *I* + *R*. Recovered individuals do not return to being susceptible as DMV confers lifelong immunity, although calves from immune mothers lose their passive immunity after a few months (Van Bressem et al., 2014 and references therein). Therefore, the birth rate (*b*) of recovered individuals was added to Equation 1; a temporal lag for immunity loss in calves could be incorporated into future modelling approaches. The birth and death (*d*) rates of all individuals were considered dependent on total population size (*D*) and carrying capacity (*K*) (Equations 1–3 in Figure 2), two parameters that are currently unknown for this population. Note that migration of individuals within the western Mediterranean or exchange with the eastern basin (Gkafas et al., 2017; Laran & Drouot‐Dulau, 2007) could also influence the population dynamics but these factors are not considered in this model. The magnitude of such dolphin movements is at present unknown.

The estimation of birth and death rates was based on available data. Mediterranean striped dolphin females mature at the age of 12 years and produce one calf every 4 years (Calzada et al., 1996); the sex ratio of the population is approximately 1:1 (Aguilar, 2000; this study). The average life span is 32 and 28 years in females and males, respectively (Calzada et al., 1997). In the model, the death rate (*d*) theoretically encompasses all causes of mortality other than (micro/macro‐) parasite infection (Anderson & May, 1978), thus we considered a 30% additional mortality from causes other than senescence or DMV (e.g. bycatch; F. J. Aznar, unpubl. data). The initial population size (*D*) was assumed to be 60% of *K*, with all dolphins being immunologically naïve to the virus (*S*) since DMV was first reported in 1990 (Domingo et al., 1990).

Transmission was considered frequency‐dependent based on previous DMV data (Morris et al., 2015), hence the transmission term was composed of the transmission rate (*β*_*V*_), the number of susceptible individuals and the proportion of infected individuals (*I/D*) at time *t* (Equations 1–2 in Figure 2). A better model adjustment was achieved by doubling the maximum daily transmission rate (*β*_*V*_) of DMV estimated for a North Pacific population of killer whale, *Orcinus orca* (Linnaeus, 1758) (Weiss et al., 2020). Differences in transmission rate are not surprising as this parameter is affected by host, pathogen and environmental characteristics (McCallum et al., 2017); for instance, killer whales generally have smaller pod sizes than striped dolphins (see de Gómez Segura et al., 2006; Weiss et al., 2020).

The infected dolphins recovered from morbillivirus at an annual rate γ, the inverse of the mean infectious period (*γ* ^−1^). Therefore, we estimated the rate of DMV‐induced mortality (*ϕ*) based on the assumption that 70% of the infected individuals die (as viruses of this family cause mortality rates of 70%–80%; Diallo et al., 2007; see Weiss et al., 2020), thus the proportion of infected individuals that die equals *ϕ*/(*ϕ + γ*) = 0.7 and *ϕ* = 0.7*γ*/(1–0.7). To capture the observed recovery and second outbreak in 2007, we inferred that the infectious period (*γ* ^−1^) of this DMV strain should be approximately 15 days, longer than that of a DMV outbreak in the northwestern Atlantic Ocean (8.3 days, range: 7.1–10 days; Morris et al., 2015). This selected value for the infectious period falls within the range of 7–18 days estimated for modelling a morbillivirus outbreak in monk seals (Baker et al., 2017).

To account for the high uncertainty in model parameters, especially those related to the epidemiology of this DMV strain, we investigated a second scenario (Scenario B), in which DMV‐induced mortality *ϕ* was reduced by a factor of 0.15, and *γ* ^−1^ was the same as for the Atlantic strain (8.3 days; Morris et al., 2015; shorter than for Scenario A). Given the known population abundance in 2001–2003 (i.e. 15,778; de Gómez Segura et al., 2006) and the lower mortality rate in Scenario B (0.15*ϕ*), the population size before 1990 was expected to be lower than for Scenario A (Table S1).

#### 2.4.2 Epibiont‐dolphin model

The second step was to investigate the response of epibiont population trends to shifts in striped dolphin abundance according to Scenarios A and B of the SIR model. We developed a mechanistic model based on the macroparasite–host Model C in Anderson and May (1978) (Equations 4–7 in Figure 2). Model parameters were either approximated or gathered from the literature (Table S1). We only modelled the interaction of striped dolphins (*D*) with *X. globicipitis* (*X*) and *S. aequus* (*C*, for cyamid), given that the population trends of *P. balaenoptera* did not vary substantially over the years and seemed to be influenced by other key drivers, such as the availability of fish hosts and seasonality (see Sections 3 and 4). Interactions between epibiotic species were not considered in the model (see Dobson, 1985, 1990), as the studied species occupy separate microhabitats, exploit different food resources and presumably have a minor impact on host fitness (see below).

Several adjustments were applied to the model equations to better represent the system. On the one hand, we added a term that considers density‐dependent constraints on host population growth (*Δ*). The term *Δ* acts as a simplified proxy of carrying capacity (*K*) that ensures that the dolphin population is at equilibrium, except for the simulated population loss by DMV. This term was omitted in Anderson and May (1978) to simplify algebraic manipulations and clarify predictions on host growth regulation by parasites; in our case, host carrying capacity was considered relevant to explore the effect of host availability on epibiotic population trends (see also SIR model).

On the other hand, we modified the base model to account for the heterogeneities in the epibionts' biology. First, parasite‐induced host mortality (α) in natural populations was indirectly estimated by dividing the natural intrinsic growth of the host population (i.e. *b* − *d*) by mean parasite abundance sensu Anderson and May (1978). This relationship suggests that parasites with low abundances are likely to cause high host mortality (Anderson & May, 1978), but the low abundance of the ectoparasite *S. aequus* may be mainly a result of limited microhabitat availability on their smooth hosts and low fecundity (see Fraija‐Fernández et al., 2017). Moreover, this species has not been associated with any reduction of host fitness (e.g. skin damage), hence α_C_ was reduced by a factor of 10. In the case of the commensal barnacle *X. globicipitis*, α_X_ was set to zero (see Arneberg et al., 1998).

Second, we modified the model to account for within‐host reproduction in the case of *S. aequus* and production of free‐living stages by *X. globicipitis* (Models E and F in May & Anderson, 1978). Given that cyamids reproduce on the host—that is they lack swimming structures, being thus transmitted by hosts' physical contact—and that brood size is approximately 6.5 (Fraija‐Fernández et al., 2017), we can assume that a part of the brood would stay on the host (*r*) and another fraction (**𝞴**_*C*_) would attempt transmission to other striped dolphins. Unfortunately, the dispersal rate of cyamids is unknown to date and our estimation of *r* > **𝞴**_*C*_ is only based on the observed mean abundance (i.e. 1.6) despite the low fecundity of this species. Under the same premise, we assumed that 80% of the transmissive stages (**𝞴**_*C*_) successfully parasitize a new host, and their transmission would be determined by the inverse transmission efficiency constant *D*_*0 C*_, where *D*/(*D*_*0 C*_ *+ D*) is the proportion of infective stages that attach to dolphins. For *X. globicipitis*, production and mortality of the free‐living stages were assumed to occur at a rate *λ*_*X*_ and *σ*_*X*_, respectively, and they would attach to dolphins at a rate *β*_*X*_ *WD*, where *W* is the number of free‐living larvae (Equations 5–6; Table S1).

Third, both epibiotic populations would decrease at the rate of mortality of the host (*d*) and epibiont (*μ*), and parasite‐induced host mortality (*α*), if any. The two latter parameters are density‐dependent, thus they vary with the number of epibionts per host, which is in turn dependent on their distribution. This is represented by the argument (*k + 1*)/*k*, where *k* is the negative binomial distribution parameter of each species; an inverse measure of aggregation that is calculated as *m* ^2^/(*s* ^2^‐*m*), with *m* and *s* ^2^ being the observed mean and variance, respectively, of the abundance of each epibiont (Anderson & May, 1978; Fisher, 1941). We used empirical data (i.e. the 1980–2023 striped dolphin dataset) for this calculation. Lastly, *β*_*X*_ and both *k* values were modified for the simulation of the 1990 and 2007 outbreaks (Table S1).

### 2.5 Statistical software and criteria

The open‐access software Quantitative Parasitology QPweb v.1.0.15 (Reiczigel et al., 2019) was used to obtain the parameter *k*, to set CIs for the prevalence and mean intensity, and to perform unconditional exact tests and bootstrap two‐sample *t*‐tests. The R package *mgcv* (Wood, 2017) was used to develop and plot the GAMs, and the R package *deSolve* (Soetaert et al., 2010) to solve numerically the SIR and macroparasite mechanistic models.

## 3 RESULTS

### 3.1 Infection parameters

Infection parameters for the three epibiotic species in different epidemiological periods and cetacean species are shown in Table 1. In striped dolphins, the prevalence of *X. globicipitis* and *P. balaenoptera* significantly differed between the 1990 DMV outbreak and the non‐epidemic years (*p* < 0.02). In contrast, neither the prevalence of *S. aequus* (*p* > 0.3) nor the mean intensity of any epibiont species (*p* > 0.08) was found to differ between these periods.

### 3.2 Long‐term trends in epibiont occurrence: Empirical data

**FIGURE 3:**
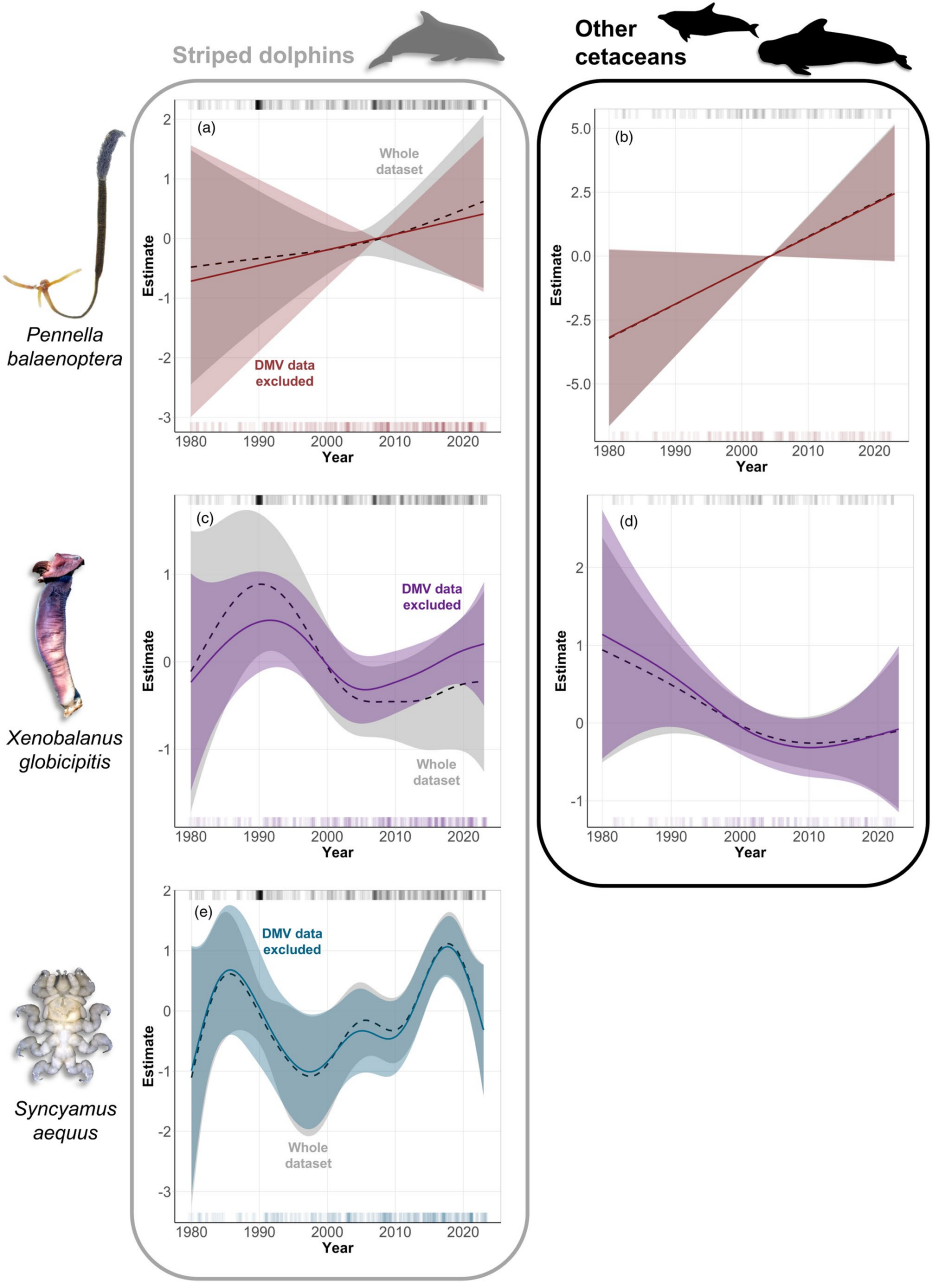
Smooth function estimates from Generalized Additive Models on the yearly trend of the occurrence of three epibiont species from cetaceans stranded along the coast of Valencia, Spain (western Mediterranean) in 1980–2023. Trends with data from the whole dataset (grey shade, dashed line) were compared with those from subsets that excluded cetaceans stranded during morbillivirus outbreaks (DMV; coloured shade, solid line). In brown, *Pennella balaenoptera* on striped dolphins, *Stenella coeruleoalba* (a) and on pooled data from four other cetacean species (see the text for details) (b). In purple, *Xenobalanus globicipitis* on striped dolphins (c) and on four other cetacean species (d); in blue, *Syncyamus aequus* on striped dolphins (e). Top and bottom ticks indicate the distribution of data points for each dataset. Regions where the confidence bands are wide and enclose the horizontal zero line indicate values where the overall pattern is not significant (Zuur et al., 2009). Figure credits: *P. balaenoptera* (Zeng & Lin, 2023), *X. globicipitis* (anonymous reviewer) and *S. aequus* (authors).

GAMs with all datasets (i.e. striped dolphins and other cetaceans, including or excluding DMV periods) failed to reveal significant long‐term changes in the occurrence of *P. balaenoptera* (Tables S2 and S3; Figure 3a,b), but they indicated a significant seasonal effect, with higher odds of occurrence in the summer (Tables S2 and S3; Figure S1C–F). In western Mediterranean striped dolphins (WMSDs), the probability of occurrence of *P. balaenoptera* increased with host length in a linear fashion (Table S2; Figure S1A). A significant effect of host length was also observed in the GAMs for the other cetaceans pooled (Table S3; Figure S1B), but note that this sample includes four species of variable body size (Table 1).

For *X. globicipitis*, the GAM for WMSDs including the whole dataset (i.e. with epizootic periods) showed a significant increase in occurrence during the 1990 DMV outbreak (grey smooth in Figure 3c and Table S2, respectively), followed by a significant drop that stabilized in the mid‐2000s and then increased; note, however, that CIs are wide (Figure 3c). These patterns were maintained after excluding the DMV data (purple smooth in Figure 3c), although no evidence for significant effects was found, and the model accounted for less variability (Table S2). The reduction in the odds of occurrence of *X. globicipitis* until the late 2000s and 2010—and subsequent recovery—was also observed in other cetacean species (Figure 3d), with a higher deviance explained but no statistical significance (Table S3). No seasonal effects were detected, and the effect of host length was only close to significance in the models with other cetacean species (Table S3). Again, the latter subset includes four cetacean species of variable size and epibiotic infection parameters (Table 1).

In the case of *S. aequus*, a significant drop in the odds of occurrence was observed after 1990, followed by a recovery until the mid‐2000s (Figure 3e; Table S2). A second (non‐significant) drop was observed around the second DMV epidemic in 2007, with occurrence significantly peaking after 2010. These patterns were consistent in the models including and excluding data from the epizootic periods. A significant effect of host length was also detected in the model with the whole dataset but not in that excluding DMV data.

### 3.3 DMV epidemiology and cascading effects on epibionts: Theoretical modelling

**FIGURE 4:**
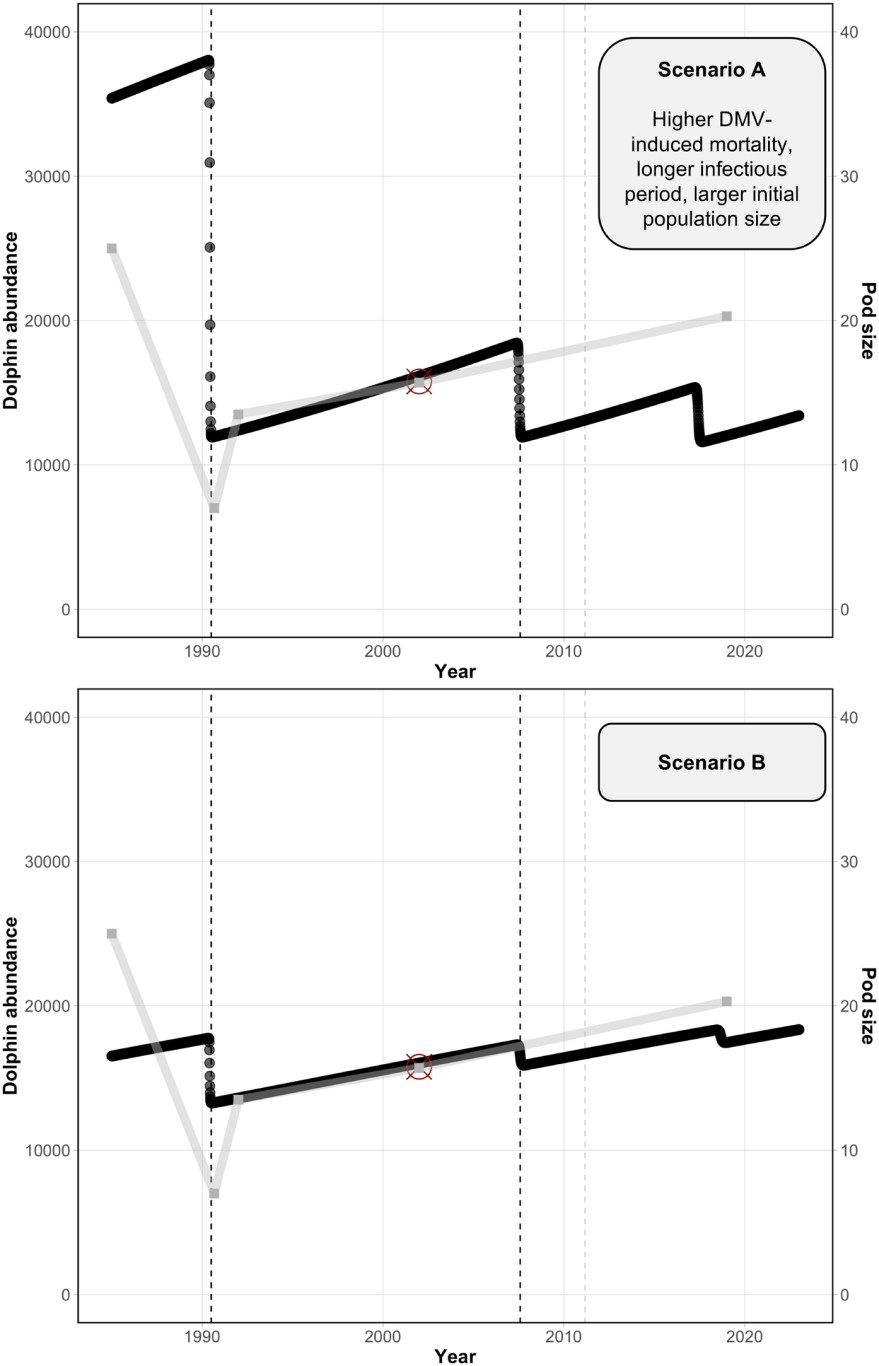
Numerical solution of a SIR model investigating the impact of dolphin morbillivirus (DMV) on the population of striped dolphins (*Stenella coeruleoalba*, black line) off Valencia, Spain (western Mediterranean). Scenario A was run with 85% higher DMV‐induced mortality among the infected individuals and a 15‐day infectious period (vs. 8.3 days in Scenario B). The grey line and right y‐axis correspond to pod size; data were gathered from the literature (see references in text). Dashed vertical lines indicate the three DMV mortalities and the red circle indicates the dolphin abundance estimated by de Gómez Segura et al. (2006).

The two proposed scenarios for the SIR model led to an estimated range of striped dolphin loss in the study area of approximately 25%–70% for the 1990 outbreak, 6%–40% for 2007 and 3%–25% for 2011 (Figure 4). Scenario A, with an 85% greater induced mortality and a longer infectious period, predicted a greater dolphin population loss (Figure 4). Nonetheless, both scenarios, A and B, led to the expected striped dolphin abundance in the study area in 2001–2003 (de Gómez Segura et al., 2006), with a population recovery of ca. 33 and 23% in 15 years (1991–2006) for Scenarios A and B, respectively (Figure 4).

**FIGURE 5:**
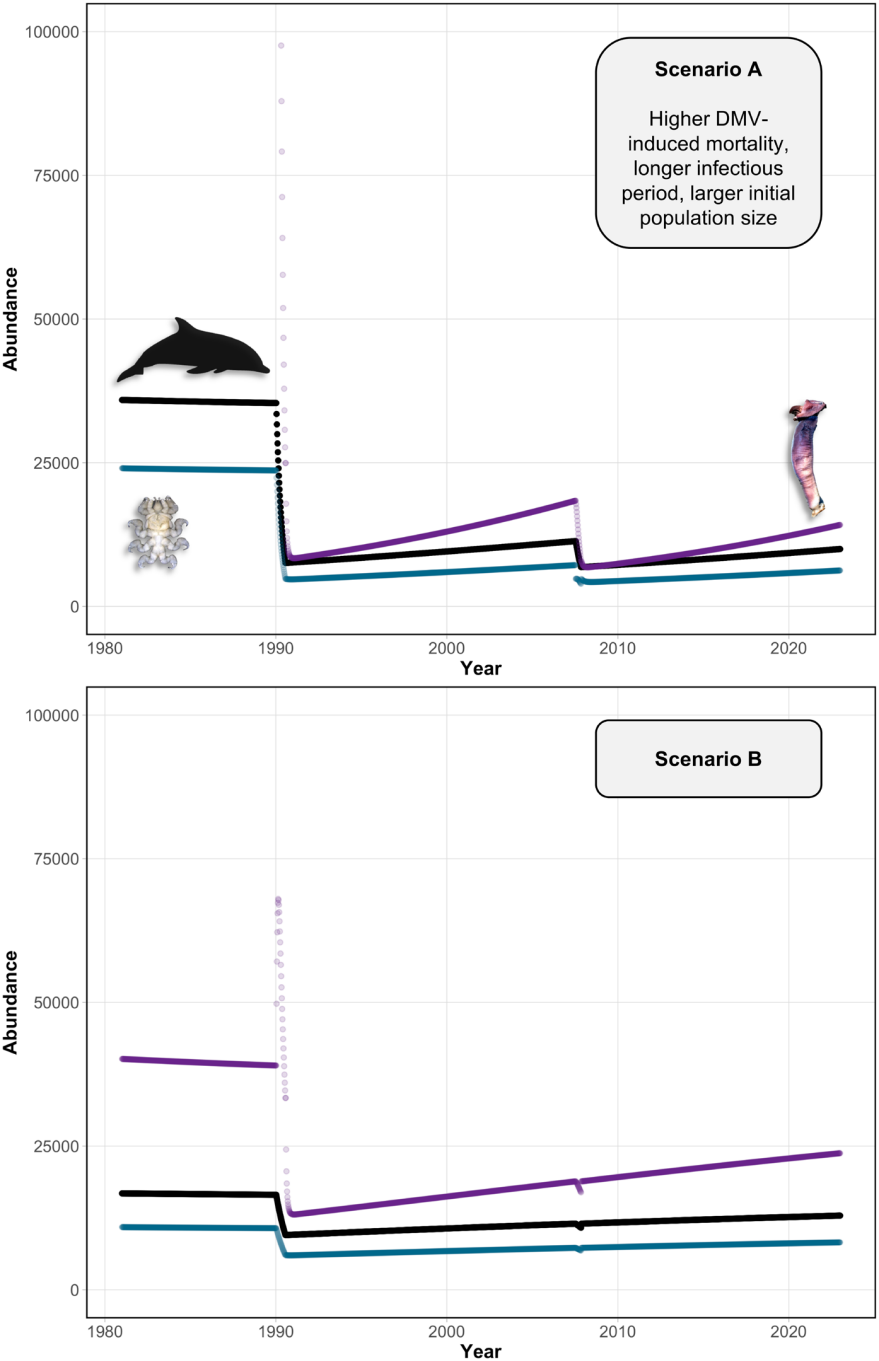
Numerical solution of a mechanistic model investigating the effect of abundance shifts in the population of the western Mediterranean striped dolphin (*Stenella coeruleoalba*, black line) on its epibiotic crustaceans *Xenobalanus globicipitis* (purple line) and *Syncyamus aequus* (blue line). Two dolphin morbillivirus (DMV) outbreaks, in 1990 and 2007, were simulated based on the dolphin population loss estimated through a previous SIR model. Scenario A considers a higher DMV‐induced mortality and longer infectious period than Scenario B. See the text and Figure 4 for details. Figure credits: *X. globicipitis* (anonymous reviewer) and *S. aequus* (authors).

In the case of *X. globicipitis*, the simulation of Scenario B better fitted the observed patterns, including the mean abundance—that is 3.6—and the predicted temporal trend, with a drop after the 1990 DMV outbreak and an indiscernible effect of the 2007 outbreak (see Figures 3 and 5). The mechanistic model failed to capture the observed mean abundance of *S. aequus*, predicting values lower than the observed mean (1.6; Figure 5). Nonetheless, the shifts of *S. aequus* mirrored host dynamics in both models, whereas *X. globicipitis* had a higher population growth rate than striped dolphins (Figure 5).

## 4 DISCUSSION

The impact of morbillivirus on dolphin populations has been a long‐standing concern in the field of cetacean conservation, especially due to the lack of quantitative data. Our integrative approach sheds new light on these dynamics. First, our analysis suggests that the populations of the epibionts *Xenobalanus globicipitis* and *Syncyamus aequus* significantly declined as a consequence of the western Mediterranean striped dolphin (WMSD) mass mortality in 1990 and recovered approximately 10 years later, when the host population had also recovered. The effect of the 2007 DMV epidemic was subtler, and the 2011 mortality did not influence epibiont abundance. Second, the impact of DMV was described with an SIR model, which could capture the 2007 epidemic based on data from the one in 1990. And third, the effects of WMSD loss on the two epibionts were captured with another mechanistic model.

### 4.1 Temporal trends in epibiotic infection

GAMs detected long‐term changes in the occurrence of two epibiotic species. The deviance explained was low (<11%; see subsection ‘Limitations and future directions’), but the trends were compatible with the expected shifts in the abundance of WMSD over time. These changes involved the two epibiotic species that are specific to cetaceans (*X*. *globicipitis* and *S*. *aequus*) and, therefore, should most likely be affected by significant population drops of WMSD, by far the most abundant cetacean in the area (i.e. Valencian region, western Mediterranean; de Gómez Segura et al., 2006; Raga & Pantoja, 2004).

The likelihood of occurrence of *X. globicipitis* significantly peaked during the 1990 morbillivirus outbreak (see also Aznar et al., 1994, 2005), then experienced a significant drop and recovered about 10 years later. We ruled out the effect of changes in host susceptibility to epibiosis as the only factor explaining this trend, as the occurrence of *X. globicipitis* consistently dropped in the other cetacean species unaffected by the DMV epidemics. During the 1990 epidemic, high numbers of *X. globicipitis* died along with their sick striped dolphin hosts. In addition to barnacle population loss, a subsequent reduction in larval production should be expected, as it has been estimated that over 80% of the larval stages of *X. globicipitis* are produced and released from striped dolphins (Aznar et al., 2016). Also, the lower density of striped dolphins would result in lower chances for barnacle larvae to attach. Consequently, the population of *X. globicipitis* would be reduced, hence the lower odds of occurrence after 1990 in all the examined cetacean species. The opposite would happen when the striped dolphin population recovered from the mortality around 2010 (Cotté et al., 2010 for the western Mediterranean; Universitat de València, unpubl. data from aerial surveys in the study area); this was observed as an increasing trend in *X. globicipitis* occurrence in this period. The impact of DMV‐induced striped dolphin mortality on *X. globicipitis* was likely buffered by the presence of ‘reservoirs’, that is cetacean species that are not affected by DMV epidemics and harbour barnacles. This could explain why the impact of the less severe 2007 epidemic and, especially, the 2011 regional mortality (i.e. shorter and with a much lower number of strandings) was not detected from *X. globicipitis* occurrence.

The occurrence trends of the cyamid *S. aequus* were similar, but with an additional subtle decline after 2007 and an increasing tendency thereafter. Changes in striped dolphin abundance could have a greater impact on this epibiont since it is much more host specific, that is it only parasitizes striped dolphins and the much scarcer common dolphins. Moreover, *S. aequus* could also be affected by changes in dolphin group composition and behaviour during and after the epidemics since it is transmitted by physical contact between hosts (Fraija‐Fernández et al., 2017). Therefore, the transmission of *S. aequus* is likely frequency‐dependent, being influenced by the proportion of hosts harbouring this cyamid in the vicinity of a potential new host (McCallum et al., 2001). Pod size was significantly lower during the 1990 outbreak (Aguilar, Borrell, et al., 1991; Aguilar, Pastor, & Forcada, 1991; Forcada et al., 1994; Forcada & Hammond, 1998) and dolphins with DMV symptoms may reduce their social interactions. Lastly, the odds of occurrence of *S. aequus* fell in the late 2010s; this is compatible with the lower striped dolphin abundance detected in the study area in recent years (Universitat de València, unpubl. data).

No long‐term trends in the occurrence of the copepod *P. balaenoptera* were detected. The population of this rather generalist parasite is likely sustained by several definitive hosts, including cetaceans and, especially, large fish, which could represent a higher proportion of the available hosts. Shifts in the abundance and susceptibility of the unknown intermediate host(s) could also play a role in the population dynamics of *P. balaenoptera* (see Brandell et al., 2021). Moreover, it seems possible that the reproduction and/or the attachment to cetaceans by *P. balaenoptera* have a seasonal pattern, as the likelihood of occurrence was greater in the summer. This finding is still compatible with the idea that immunosuppressed hosts are more susceptible to parasitization by *P. balaenoptera* (Aznar et al., 2005), but suggests that both factors could contribute to the higher prevalence observed during the 1990 epidemic. The significant positive effect of host length on the occurrence of *P. balaenoptera* might be a synergy between higher contact rate with larger hosts and greater DMV‐derived mortality in adult dolphins during the 1990 outbreak (Aguilar & Raga, 1993)—the latter hypothesis also applies to *S. aequus*. For the dataset with other cetacean species spanning a range of sizes (i.e. from common dolphins to pilot whales) and ecological niches, the effect of host length could be masking their different susceptibility to epibiosis (e.g. lower prevalence of *P. balaenoptera* in the more coastal bottlenose dolphins; Fraija‐Fernandez et al., 2018).

In line with our initial hypothesis, the differences in the life cycle and host specificity of the studied epibiotic species shape their use and resolution as indicators. In particular, the host‐specific *X. globicipitis* and, especially, *S. aequus* seem capable of tracing the abundance shifts of striped dolphins and are, therefore, able to detect population decline and recovery after DMV outbreaks. Nonetheless, the impact of DMV could have been higher than what could be detected in a non‐random sample of stranded cetaceans. Altogether, the relationship between the dynamics of a host and its epifauna is largely influenced by the life cycle of the epibionts, including transmission and reliance on intermediate hosts, and the degree of host specificity. Future surveys analysing the epibiotic occurrence in live animals at sea will allow for potentially assessing the degree of discordance with estimates from strandings.

As opposed to the occurrence (a proxy for the prevalence), the intensity of the epibionts did not significantly change during the outbreak. It has been suggested that several regulating mechanisms operate between and within parasite–host systems, given that parasite prevalence and abundance do not always correlate to host abundance (e.g. Stanko et al., 2002, 2006). In the case of *X. globicipitis* and *P. balaenoptera*, it seems likely that the spatial distribution of the free‐living stages is patchy (see Anderson & Gordon, 1982; Foufopoulos et al., 2022), with higher densities of transmissive stages in areas where eggs of *X. globicipitis* and *P. balaenoptera* have been released from cetaceans, or gravid *P. balaenoptera* have left their intermediate hosts (e.g. Arroyo et al., 2002; Dreyer et al., 2020). One may think that dolphins with severe DMV could harbour higher epibiotic loads due to an increased contact rate due to slower swimming and to immunological susceptibility (see above); however, the patchy distribution of infective stages in the environment facilitates that healthy hosts could also contact high densities of infective stages in a particular time and location. For *S. aequus*, intensity may be mostly regulated by its own carrying capacity at the infrapopulation (within host) level, rather than host health or abundance. For this species, microhabitat availability and mate finding seem major drivers of infrapopulation growth (Fraija‐Fernández et al., 2017).

### 4.2 Mechanistic models

The patterns discussed above reveal a clear but complex relationship between the abundance of the hosts and that of epibionts. These interspecific associations were described through a series of equations for the two species that are more influenced by the population dynamics of the WMSDs, that is *X. globicipitis* and *S. aequus*. To explore the observed changes in epibiosis due to the epidemics, we first modelled two putative scenarios for the DMV‐dolphin dynamics through an SIR model and used the estimated population loss by DMV to assess the isolated effect of host shifts on the epibionts.

#### 4.2.1 Susceptible‐infected‐recovered (SIR) model

Both scenarios of the SIR model captured the 1990 and 2007 DMV epidemics, their interepizootic time interval, the greater severity of the first outbreak, the estimated abundance in the study area in 2001–2003 (de Gómez Segura et al., 2006), and the abundance shifts expected from pod size estimates (Aguilar, Borrell, et al., 1991; Aguilar, Pastor, & Forcada, 1991; Forcada et al., 1994 and references therein; de Gómez Segura et al., 2006; Forcada & Hammond, 1998; Gannier, 2005; Paradell et al., 2023). None of the scenarios could reproduce the milder 2011 mortality; the epidemiological parameters of this event could have been very different from the previous ones—that is lower mortality and less severe gross and histopathologic lesions compared to the two previous outbreaks (see Rubio‐Guerri et al., 2013).

Scenario A, which suggests that almost 70% of the striped dolphin population died during the 1990 epidemic, is more compatible with the recent lower abundance in the study area versus that around 2010 (Universitat de València, unpubl. data). This population loss estimate is much higher than the observed 30% reduction in pod size, but the latter could be a conservative estimate of dolphin population loss considering regrouping of the surviving individuals (Aguilar & Raga, 1993; Forcada et al., 1994; see also subsection ‘Limitations and future directions’). In fact, other morbilliviruses such as peste des petits ruminants virus (PPRV) may kill 70%–80% of infected hosts from immunologically naive populations (Diallo et al., 2007), such as Mediterranean striped dolphins prior to being exposed to DMV in the 1990 epidemic (Domingo et al., 1990; Raga et al., 2008).

For the interepizootic period (i.e. 1991–2006), models suggest that striped dolphins in the Valencian region recovered by ca. 23% and 33% for Scenarios B and A, respectively. These values are somewhat lower than the 43% recovery observed for the same time period in waters surrounding the Balearic Islands (Cotté et al., 2010). Note, however, that some degree of heterogeneity could be expected in the epidemiology of different regions; but, unfortunately, no other abundance estimates are available for this period. Also, the model does not take into account potential non‐linear relationships, which could account for, for example a higher population recovery rate. Altogether, these models show that a broad range of input parameters could lead to reasonable outputs, emphasizing the need of obtaining better parameter estimates for DMV.

#### 4.2.2 Epibiont‐dolphin model

Under both model scenarios, the abundance of *S. aequus* mirrored that of striped dolphins in the macroparasite–host model. Mean abundance was lower than that observed in stranded dolphins, but these do not represent a random subsample of the population (see below). In the case of *X. globicipitis*, both scenarios trace the abundance drop after 1990; in Scenario A, the 2007 DMV outbreak also resulted in a reduction of the barnacle population, while the effect was very subtle under Scenario B and mean abundance is closer to the observed value. Even if Scenario B better represents the GAM trends, these are conservative due to the nature of the data (i.e. strandings likely biased towards higher epibiont prevalence; see ‘Limitations and future directions’ below), thus the impact of the second outbreak on the population of *X. globicipitis* should not be neglected. The uncertainties around the actual impact of DMV on the striped dolphin population, as well as the gaps of information on the reproduction and transmission of these two epibionts, limit the predictive power of the mechanistic models. Nonetheless, this theoretical approach illustrates the link between the population dynamics of both epibionts and that of striped dolphins and the cascading effects of viral outbreaks on epibiotic populations.

### 4.3 Limitations and future directions

#### 4.3.1 Stranding data

Given the difficulties of monitoring cetaceans at sea, most inferences to date rely on stranding data (Van Bressem et al., 2014 and references therein), a valuable and generally accessible source of information (e.g. Peltier et al., 2014). Nonetheless, some potential biases should be considered when examining stranding data. First, only a low percentage of the dead cetaceans strand (Aguilar & Raga, 1993; Epperly et al., 1996; Moore et al., 2020; Peltier et al., 2012, 2016), and the number of strandings is not only determined by cetacean abundance and mortality but also by other factors like buoyancy, currents or reporting rates (e.g. Meager & Sumpton, 2016; Peltier et al., 2014; Saavedra et al., 2017). Therefore, stranding events may underestimate the actual death toll and epidemiology of DMV epidemics; hence, outbreaks investigated from stranding data are referred to as ‘partially observed epidemic processes’ (Morris et al., 2015). Second, stranded individuals are not a random subsample of the population. Mortality risk varies with ontogeny, so the study of demographics is partly hindered (Perrin et al., 2002). Third, a proportion of the stranded animals have suffered processes of disease, thus not being a clear representation of the health status or other biological parameters of the overall population.

These biases pervade the present study. A recent survey involving stranded WMSDs in the study area determined that ca. three‐quarters of stranded dolphins had died from infectious disease (F. J. Aznar, unpubl. data). If sick animals showed impaired immune function and/or swimming performance, their epibiotic prevalence could be abnormally high (Aznar et al., 1994, 2005; Vecchione & Aznar, 2014) and, therefore, any long‐term change of epibiotic infections in the wild would be blurred in a sample of stranded dolphins (again, a plausible explanation for the low deviance explained in the GAMs). However, even under this conservative scenario, GAMs detected long‐term changes in the occurrence of two epibiotic species.

#### 4.3.2 Input data for the mechanistic models

For the mechanistic models, the assessment of model reliability and the estimation of some unknown parameters were based on (1) abundance estimates in the study area (de Gómez Segura et al., 2006) and in the larger western Mediterranean (Cotté et al., 2010; Forcada & Hammond, 1998) and (2) data on pod size at the same two spatial scales (Aguilar, Borrell, et al., 1991; Aguilar, Pastor, & Forcada, 1991; Forcada et al., 1994 and references therein; de Gómez Segura et al., 2006; Forcada & Hammond, 1998; Paradell et al., 2023). The latter comparison should be treated with caution, given that group size does not necessarily correlate with population abundance in dolphins (Hammond et al., 2017), and that regrouping has been observed after a sharp population drop to improve survival and reproductive capabilities (Aguilar & Raga, 1993; Forcada et al., 1994).

The model input parameters related to DMV epidemiology (i.e. transmission rate, mortality, infectious period) were based on those obtained during morbillivirus outbreaks in the Atlantic and Pacific oceans (Morris et al., 2015; Weiss et al., 2020). This approach was used given the lack of epidemiological data for Mediterranean DMV. Besides, it also carries some uncertainty as these parameters may vary between outbreaks (i.e. given the different recorded mortality of the three striped dolphin outbreaks described here) and host species (see Van Elk et al., 2014). Altogether, this should be regarded as a preliminary model whose predictive capacity will improve when more reliable input parameters are obtained through continued monitoring of striped dolphins (see Van Bressem et al., 2014) and studies on DMV genome and infection in cell cultures (Jo et al., 2018). Monitoring programs should gather data on dolphin abundance, serology (to estimate, e.g. the proportion of immune individuals), and toxicology—high levels of immunotoxic pollutants have been associated with increased susceptibility to DMV in 1990 (Borrell & Aguilar, 1991) but not in 2007 (Castrillon et al., 2010).

## 5 CONCLUSIONS

The high dependence of the populations of *X. globicipitis* and *S. aequus* on Mediterranean striped dolphins in the western Mediterranean (see Aznar et al., 2016) makes them reliable proxies for investigating shifts in the abundance of this cetacean. To date, the predictive potential of the mechanistic models (i.e. both SIR and epibiont‐dolphin) is hindered by the numerous uncertainties regarding the abundance of WMSDs, the epidemiology of each DMV outbreak and the biology of the two epibiotic species (e.g. loss of larval stages of *X. globicipitis* or transmission rate of *S. aequus*). Accordingly, the indicator potential of these epibionts is now oriented towards detecting host abundance shifts and not towards accurately quantifying host population changes. Nonetheless, the presented theoretical modelling framework serves as a steppingstone for future studies that use better input parameters and highlights the importance of considering the population dynamics of host‐microparasite‐macroparasite communities (vs. single‐parasite models that can predict different responses; Fenton, 2008). When long/medium‐term datasets are available, the temporal trends of *X. globicipitis* and *S. aequus* can be used as a tool for investigating the population dynamics of their hosts.

## AUTHOR CONTRIBUTIONS

Sofía Ten, Gates Dupont, Andy P. Dobson and Francisco Javier Aznar conceived the ideas and designed methodology; Sofía Ten, Juan Antonio Raga and Francisco Javier Aznar collected the data; Sofía Ten, Gates Dupont and Francisco Javier Aznar analysed the data; Sofía Ten led the writing of the manuscript; Juan Antonio Raga and Francisco Javier Aznar acquired funding. All authors contributed critically to the drafts and gave final approval for publication.

## CONFLICT OF INTEREST STATEMENT

The authors declare there are no conflicts of interest.

## Supporting information


**Figure S1.** Results of Generalized Additive Models showing the partial effects of ‘host length’ and ‘season’ (A–D) and the combined effects of the interaction ‘season: year’ (E–H) on the occurrence of the parasite *Pennella balaenoptera* on western Mediterranean cetaceans. Models were run separately for striped dolphins (*Stenella coeruleoalba*, left column: A, C, E, G) and other four cetacean species (right column: B, D, F, H). In A–D, trends with data from whole datasets (grey shade, dashed line) were compared with those from subsets that excluded cetaceans stranded during morbillivirus outbreaks (DMV; coloured shade, solid line). Similarly, graphs E, F correspond to data from whole datasets, whereas G, H belong to datasets excluding data from DMV outbreaks.
**Table S1.** Parameters of the SIR and dolphin‐epibiont mechanistic models for western Mediterranean striped dolphin morbillivirus (DMV) and epibiotic crustaceans *Xenobalanus globicipitis* and *Syncyamus aequus*. For the SIR model, values correspond to those of Scenario A, and those modified for Scenario B are shown in square brackets. See the text for details of parameter estimates and references.
**Table S2.** Backward selection of binomial Generalized Additive Models based on the Akaike Information Criterion with small sample correction (AICc). Models investigated temporal trends in the likelihood of occurrence of three epibiotic species from striped dolphins, *Stenella coeruleoalba*, stranded in the western Mediterranean; also accounting for host length and sex and season. Other values for model evaluation include the *R* ^2^‐adjusted coefficient and the percentage of deviance explained. The null model only included the intercept.
**Table S3.** Backward selection of binomial Generalized Additive Models based on the lowest value of Akaike information criterion with small sample correction (AICc). Models investigated temporal trends in the likelihood of occurrence of two epibiotic species from four species of odontocetes (48 bottlenose dolphins, *Tursiops truncatus*; 30 Risso's dolphins, *Grampus griseus*; 20 common dolphins, *Delphinus delphis*; and 9 long‐finned pilot whales, *Globicephala melas*) stranded in the western Mediterranean; also accounting for host length and sex and season. Other values for model evaluation include the *R* ^2^‐adjusted coefficient and the percentage of deviance explained. The null model included only the intercept.

## Data Availability

Data available from the Dryad Digital Repository: https://doi.org/10.5061/dryad.z8w9ghxtf (Ten et al., 2026).
