# Supplementary material for: Long‐term trends of epibionts reflect Mediterranean striped dolphin abundance shifts caused by morbillivirus epidemics
Source: J Anim Ecol. 2026 Feb 6;95(3):553–69. doi: 10.1111/1365-2656.70216 (PMC12957715; doi:10.1111/1365-2656.70216)
Supplement: Supplementary file 1 — Figure S1. Results of Generalized Additive Models showing the partial effects of ‘host length’ and ‘season’ (A–D) and the combined effects of the interaction ‘season: year’ (E–H) on the occurrence of the parasite Pennella balaenoptera on western Mediterranean cetaceans. Models were run separately for striped dolphins (Stenella coeruleoalba, left column: A, C, E, G) and other four cetacean species (right column: B, D, F, H). In A–D, trends with data from whole datasets (grey shade, dashed line) were compared with those from subsets that excluded cetaceans stranded during morbillivirus outbreaks (DMV; coloured shade, solid line). Similarly, graphs E, F correspond to data from whole datasets, whereas G, H belong to datasets excluding data from DMV outbreaks. Table S1. Parameters of the SIR and dolphin‐epibiont mechanistic models for western Mediterranean striped dolphin morbillivirus (DMV) and epibiotic crustaceans Xenobalanus globicipitis and Syncyamus aequus. For the SIR model, values correspond to those of Scenario A, and those modified for Scenario B are shown in square brackets. See the text for details of parameter estimates and references. Table S2. Backward selection of binomial Generalized Additive Models based on the Akaike Information Criterion with small sample correction (AICc). Models investigated temporal trends in the likelihood of occurrence of three epibiotic species from striped dolphins, Stenella coeruleoalba, stranded in the western Mediterranean; also accounting for host length and sex and season. Other values for model evaluation include the R 2‐adjusted coefficient and the percentage of deviance explained. The null model only included the intercept. Table S3. Backward selection of binomial Generalized Additive Models based on the lowest value of Akaike information criterion with small sample correction (AICc). Models investigated temporal trends in the likelihood of occurrence of two epibiotic species from four species of odontocetes (48 bottlen [file JANE-95-553-s001.docx]

**Supplementary Material**

Ten, S., Dupont, G., Raga, J. A., Dobson, A. P., Aznar, F. J. (2026). Long-term trends of epibionts reflect Mediterranean striped dolphin abundance shifts caused by morbillivirus epidemics. *Journal of Animal Ecology*. doi: 10.1111/1365-2656.70216

**Supplementary Figure 1.** Results of Generalized Additive Models showing the partial effects of ‘host length’ and ‘season’ (A-D) and the combined effects of the interaction ‘season:year’ (E-H) on the occurrence of the parasite *Pennella balaenoptera* on western Mediterranean cetaceans. Models were run separately for striped dolphins (*Stenella coeruleoalba*, left column: A, C, E, G) and other four cetacean species (right column: B, D, F, H). In A-D, trends with data from whole datasets (grey shade, dashed line) were compared with those from subsets that excluded cetaceans stranded during morbillivirus outbreaks (DMV; colored shade, solid line). Similarly, graphs E-F correspond to data from whole datasets, whereas G-H belong to datasets excluding data from DMV outbreaks.

**Supplementary Table 1.** Parameters of the SIR and dolphin-epibiont mechanistic models for western Mediterranean striped dolphin morbillivirus (DMV) and epibiotic crustaceans *Xenobalanus globicipitis* and *Syncyamus aequus*. For the SIR model, values correspond to those of Scenario A, and those modified for Scenario B are shown in square brackets. See the text for details of parameter estimates and references.

| **Parameter** | **Value** | **Description** |
| --- | --- | --- |
| **K** | 60’000 [28’000] | Dolphin carrying capacity; adjusted to expected model output |
| **b** | 0.078 | Dolphin birth rate, births/year/dolphin; estimated from Calzada et al. (1996) and Aguilar (2000) |
| **d** | 0.04 | Dolphin death rate, deaths/year/dolphin, by senescence (see Calzada et al., 1997) and other causes of mortality different from DMV (unpubl. data on other causes of death) |
| **β_V_** | 197.1 | DMV transmission rate, transmissions/year/dolphin; see Weiss et al. (2020) |
| **γ_V_** | 24.3 [43.9] | DMV recovery rate, recoveries/year/infected Dolphin; given an infectious period of 8.3 days (Morris et al., 2015), applicable for Scenario B [*vs.* 15 days in Scenario A] |
| **ϕ** | 56.8 [14.2] | DMV-induced death rate, deaths/year/infected dolphin; estimation based on Weiss et al. (2020), with a reduction factor of 0.15 for Scenario B |
| 𝞴_X_ | 15’190 | Rate of production of infective stages of *X. globicipitis*, i.e., number of eggs (Alonso, unpubl.) |
| **σ**_X_ | 365/8 | Rate of loss of transmissive stages of *X. globicipitis* (losses/year/larva) due to death or other processes that prevent attachment to hosts. Inferred from the duration of larval development at *in vitro* conditions (i.e., 8 days; Dreyer et al., 2020), thus likely underestimated as other sources of mortality at sea were not considered. |
| **D_0_** _X_ | (D - 0.002D) / 0.002 | Inverse transmission efficiency constant of *X. globicipitis*, where D/(D_0_ + D) is the proportion of infective stages that attach to dolphins; this estimate yielded the closest value to the observed abundance (i.e., approximately 3) and can be interpreted as 0.2% of the cyprids attach to hosts. This parameter was increased a 10% during the two simulated outbreaks; see main text for details. |
| **β**_X_ | σ_X_ / D_0 X_ | Transmission rate of infective stages of *X. globicipitis*, i.e., cyprid attachment. At equilibrium, D_0 X_ = σ_X_ / β_X_ (May and Anderson, 1978). Increased by a 10% during the 1990 outbreak to account for higher host susceptibility (Aznar et al., 1994, 2005); this pattern was not detected during the 2007 outbreak (Fig. 3C). |
| 𝞵_X_ | 1/0.75 | Mortality rate of *X. globicipitis*, given that the life span was estimated to be <1 year (see Flach et al., 2021) and that spawning may occur every 6 months (Van Waerebeek et al., 1993). In addition to losses due to senescence, we assume that this parameter encompasses other factors limiting epibiotic population growth (e.g., microhabitat availability), which could be alternatively included as a separate term. |
| 𝞪_X_ | 0 | Host mortality induced by *X. globicipitis* |
| **k**_X_ | 0.11 | Negative binomial distribution parameter of *X. globicipitis* calculated from empirical data; k_X_= 0.27 was used for the 1990 outbreak simulation (Aznar et al., 2005) |
| **r** | 6.5 – 𝞴_C_ | Rate of parasite births of *S. aequus* within the host. Brood size is approximately 6.5 (Fraija-Fernández et al., 2017), with an unknown fraction staying in the host (r) and another fraction attempting transmission to other striped dolphins (𝞴_C_). |
| 𝞴_C_ | 2 | Number of transmissive individuals of *S. aequus*; estimation (see above) |
| **D_0_** _C_ | (D - 0.8D) / 0.8 | Inverse transmission efficiency constant, where D/(D_0_ + D) is the proportion of infective stages that attach to dolphins. We assumed that 80% of the transmissive stages (𝞴 _S_) successfully parasitize a new host. |
| 𝞪_C_ | 0.003 | Parasite-induced host mortality of *S. aequus*; estimated *sensu* Anderson and May (1978) and reduced to a 10% (see the text). |
| 𝞵_C_ | 1 | Mortality rate of *S. aequus*; a one-year long life cycle is assumed for *Cyamus* spp. (Leung, 1976; Ólafsdóttir and Shinn, 2013) |
| **k**_C_ | 0.12 | Negative binomial distribution parameter of *S. aequus* calculated from empirical data; k_X_ was similar during the 1990 and 2007 outbreaks |

**Supplementary Table 2.** Backward selection of binomial Generalized Additive Models based on the Akaike Information Criterion with small sample correction (AICc). Models investigated temporal trends in the likelihood of occurrence of three epibiotic species from striped dolphins, *Stenella coeruleoalba*, stranded in the western Mediterranean; also accounting for host length and sex, and season. Other values for model evaluation include the R^2^-adjusted coefficient and the percentage of deviance explained. The null model only included the intercept. Asterisks indicate significant smooth terms: *, p-value<0.05; **, <0.01; ***, <0.001; and non-significant terms with p-value<0.10 are underlined. Models represented graphically are in bold; see main text.

| **Response variable** | **Dataset** | **Parameters** | **ΔAICc** | **AICc** | **Adj-R^2^** | **Dev (%)** |
| --- | --- | --- | --- | --- | --- | --- |
| Presence/absence of  *Pennella balaenoptera* | Whole dataset  (N= 352 striped dolphins) | s(length)** + s(season)*** + s(year, by= season)** + sex | - | 324.9 | 0.1110 | 13.1 |
|  |  | **s(year) + s(length)** + s(season)*** + s(year, by= season)** + sex** | 1.1 | 326.0 | 0.1110 | 13.6 |
|  |  | s(year) + s(length)** + s(season)** + s(year, by= season)** | 1.7 | 326.6 | 0.1090 | 12.3 |
|  |  | Intercept | 33.8 | 358.7 | 0 | <0.0 |
|  |  |  |  |  |  |  |
|  | DMV excluded  (N= 269) | s(length)* + s(season)** + s(year, by= season)** + sex | - | 232.3 | 0.0994 | 12.8 |
|  |  | **s(year) + s(length)* + s(season)** + s(year, by= season)** + sex** | 1.7 | 234.0 | 0.0961 | 12.9 |
|  |  | Intercept | 19.0 | 251.3 | 0 | 0 |
|  |  |  |  |  |  |  |
| Presence/absence of  *Xenobalanus globicipitis* | Whole dataset  (N= 342) | **s(year) + s(length) + s(season) + s(year, by= season**) | - | 460.5 | 0.0446 | 5.0 |
|  |  | s(year) + s(season) + s(year, by= season) | 0.8 | 461.3 | 0.0356 | 3.9 |
|  |  | s(year)* + s(length) + s(season) | 1.0 | 461.5 | 0.0325 | 3.5 |
|  |  | s(year) + s(length) + s(season) + s(year, by= season) + sex | 2.8 | 463.3 | 0.0428 | 5.3 |
|  |  | Intercept | 6.4 | 466.9 | <0.0000 | 0 |
|  |  |  |  |  |  |  |
|  | DMV excluded  (N= 262) | s(length) + s(season)* + sex | - | 350.6 | 0.02110 | 2.9 |
|  |  | **s(year) + s(length) + s(season)* + sex** | 0.2 | 350.8 | 0.0346 | 4.9 |
|  |  | Intercept | 0.8 | 351.4 | <0.0000 | <0.0 |
|  |  | s(year) + s(season) + s(year, by= season) + sex | 1.2 | 351.8 | 0.0320 | 4.7 |
|  |  | s(year) + s(length) + s(season) + s(year, by= season) + sex | 3.0 | 353.6 | 0.0315 | 5.1 |
|  |  |  |  |  |  |  |
| Presence/absence of  *Syncyamus aequus* | Whole dataset  (N= 337) | s(year)** + s(length)* | 0 | 367.6 | 0.0897 | 9.9 |
|  |  | **s(year)** + s(length)* + s(season)** | 1.1 | 368.7 | 0.0897 | 9.9 |
|  |  | s(year)** + s(length)* + s(season) + s(year, by= season) | 2.5 | 370.1 | 0.0949 | 10.6 |
|  |  | s(year)** + s(length)* + s(season) + s(year, by= season) + sex | 6.5 | 374.1 | 0.0897 | 10.6 |
|  |  | Intercept | 21.5 | 389.1 | 0 | <0.0 |
|  |  |  |  |  |  |  |
|  | DMV excluded  (N= 259) | **s(year)** + s(length) + s(season)** | - | 287.4 | 0.0962 | 10.5 |
|  |  | **s(year)** + s(length)** | 0 | 287.4 | 0.0962 | 10.5 |
|  |  | s(year)** | 1.0 | 288.4 | 0.0838 | 9.0 |
|  |  | s(year)** + s(length) + s(season) + s(year, by= season) | 2.5 | 289.9 | 0.0947 | 11.0 |
|  |  | s(year)** + s(length) + s(season) + s(year, by= season) + sex | 6.0 | 293.4 | 0.0905 | 11.3 |
|  |  | Intercept | 14.9 | 302.3 | <0.0000 | <0.0 |

**Supplementary Table 3.** Backward selection of binomial Generalized Additive Models based on the lowest value of Akaike Information Criterion with small sample correction (AICc). Models investigated temporal trends in the likelihood of occurrence of two epibiotic species from four species of odontocetes (48 bottlenose dolphins, *Tursiops truncatus*; 30 Risso’s dolphins, *Grampus griseus*; 20 common dolphins, *Delphinus delphis*; and 9 long-finned pilot whales, *Globicephala melas*) stranded in the western Mediterranean; also accounting for host length and sex, and season. Other values for model evaluation include the R^2^-adjusted coefficient and the percentage of deviance explained. The null model included only the intercept. Asterisks indicate significant smooth terms, with p-value<0.05; **, <0.01; ***, <0.001; non-significant terms with p-value<0.10 are underlined. Models represented graphically are in bold; see main text.

| **Response variable** | **Dataset** | **Parameters** | **ΔAIC** | **AIC** | **Adj-R^2^** | **Dev (%)** |
| --- | --- | --- | --- | --- | --- | --- |
| Presence/absence of  *Pennella balaenoptera* | Whole dataset  (N= 106 cetaceans) | s(year) + s(length)*** + s(season) + s(year, by= season)** | - | 102.5 | 0.1940 | 20.7 |
|  |  | **s(year) + s(length)*** + s(season) + s(year, by= season)* + sex** | 1.7 | 104.2 | 0.1940 | 21.1 |
|  |  | Intercept | 15.3 | 117.8 | 0 | <0.0 |
|  |  |  |  |  |  |  |
|  | DMV excluded  (N= 104) | s(year) + s(length)*** + s(season) + s(year, by= season)* | - | 102.1 | 0.1900 | 20.2 |
|  |  | **s(year) + s(length)*** + s(season) + s(year, by= season)* + sex** | 1.7 | 103.8 | 0.1890 | 20.7 |
|  |  | Intercept | 13.0 | 116.8 | <0.0000 | <0.0 |
|  |  |  |  |  |  |  |
| Presence/absence of  *Xenobalanus globicipitis* | Whole dataset  (N= 103) | s(length) | - | 136.5 | 0.0879 | 9.2 |
|  |  | **s(year) + s(length) + s(season)** | 0.4 | 136.9 | 0.1070 | 11.3 |
|  |  | **s(year) + s(length)** | 0.4 | 136.9 | 0.1070 | 11.3 |
|  |  | s(year) + s(length) + s(season) + s(year, by= season) | 2.9 | 139.4 | 0.1080 | 13.0 |
|  |  | s(year) + s(length) + s(season) + s(year, by= season) + sex | 5.3 | 141.8 | 0.0988 | 13.0 |
|  |  | Intercept | 7.5 | 144.0 | 0 | 0 |
|  |  |  |  |  |  |  |
|  | DMV excluded  (N= 101) | **s(year) + s(length) + s(season)** | - | 132.7 | 0.1280 | 12.8 |
|  |  | s**(year) + s(length)** | 0 | 132.7 | 0.1280 | 12.8 |
|  |  | s(length)* | 0.5 | 133.2 | 0.0984 | 9.9 |
|  |  | s(year) + s(length) + s(season) + s(year, by= season) | 2.8 | 135.5 | 0.1270 | 14.4 |
|  |  | s(year) + s(length) + s(season) + s(year, by= season) + sex | 5.2 | 137.9 | 0.1170 | 14.4 |
|  |  | Intercept | 8.2 | 141.6 | <0.0000 | <0.0 |
